# Enhanced Contractive Tension and Upregulated Muscarinic Receptor 2/3 in Colorectum Contribute to Constipation in 6-Hydroxydopamine-Induced Parkinson’s Disease Rats

**DOI:** 10.3389/fnagi.2021.770841

**Published:** 2021-12-23

**Authors:** Xiao-Li Zhang, Xiao-Hui Zhang, Xiao Yu, Li-Fei Zheng, Xiao-Yan Feng, Chen-Zhe Liu, Zhu-Sheng Quan, Yue Zhang, Jin-Xia Zhu

**Affiliations:** ^1^Department of Physiology and Pathophysiology, School of Basic Medical Sciences, Capital Medical University, Beijing, China; ^2^Artificial Liver Treatment Center, Beijing Youan Hospital, Capital Medical University, Beijing, China

**Keywords:** PD, Parkinson’s disease, constipation, sacral parasympathetic nucleus, acetylcholine, colorectal dysmotility

## Abstract

Constipation and defecatory dysfunctions are frequent symptoms in patients with Parkinson’s disease (PD). The pathology of Lewy bodies in colonic and rectal cholinergic neurons suggests that cholinergic pathways are involved in colorectal dysmotility in PD. However, the underlying mechanism is unclear. The aim of the present study is to examine the effect of central dopaminergic denervation in rats, induced by injection 6-hydroxydopamine into the bilateral substania nigra (6-OHDA rats), on colorectal contractive activity, content of acetylcholine (ACh), vasoactive intestinal peptide (VIP) and expression of neural nitric oxide synthase (nNOS) and muscarinic receptor (MR). Strain gauge force transducers combined with electrical field stimulation (EFS), gut transit time, immunohistochemistry, ELISA, western blot and ultraperformance liquid chromatography tandem mass spectrometry were used in this study. The 6-OHDA rats exhibited outlet obstruction constipation characterized by prolonged transit time, enhanced contractive tension and fecal retention in colorectum. Pretreatment with tetrodotoxin significantly increased the colorectal motility. EFS-induced cholinergic contractions were diminished in the colorectum. Bethanechol chloride promoted colorectal motility in a dose-dependent manner, and much stronger reactivity of bethanechol chloride was observed in 6-OHDA rats. The ACh, VIP and protein expression of nNOS was decreased, but M_2_R and M_3_R were notably upregulated in colorectal muscularis externa. Moreover, the number of cholinergic neurons was reduced in sacral parasympathetic nucleus (SPN) of 6-OHDA rats. In conclusion, central nigrostriatal dopaminergic denervation is associated with decreased cholinergic neurons in SPN, decreased ACh, VIP content, and nNOS expression and upregulated M_2_R and M_3_R in colorectum, resulting in colorectal dysmotility, which contributes to outlet obstruction constipation. The study provides new insights into the mechanism of constipation and potential therapeutic targets for constipation in PD patients.

## Introduction

Gastrointestinal (GI) symptoms, especially constipation and defecatory dysfunctions, are very common in the preclinical stage of Parkinson’s disease (PD) (Knudsen et al., 2017; Chen et al., 2020). Clinical diagnosis of PD is commonly dependent on cardinal motor symptoms (Tolosa et al., 2021), which occur when the loss of dopaminergic neurons reaches approximately 80% in the striatum and 40–50% or more in the substantia nigra (SN) (Fearnley and Lees, 1991; Johnson et al., 2018). Increasing evidence suggests that PD starts in the gut, with retrograde transmission of misfolded alpha-synuclein spreading from the enteric nervous system (ENS) to higher brain centers along vagal afferents (Braak et al., 2003; Holmqvist et al., 2014). This evidence may indicate that constipation in PD originates from dysfunction of the ENS. On the other hand, constipation is very closely correlated with nigrostriatal dopaminergic denervation in early PD patients and associated with low SN neuron density independent of the presence of Lewy bodies (Petrovitch et al., 2009; Hinkle et al., 2018). In fact, there is direct evidence that nigral pathology may exacerbate GI dysfunction. We have reported that rats with 6-hydroxydopamine microinjected in the bilateral SN (6-OHDA rats) exhibit delayed gastric emptying (Zheng et al., 2011, 2014), impaired duodenal mucus secretion (Yan et al., 2021) and outlet obstruction constipation (Zhang et al., 2015). Moreover, dopaminergic neurons in the SN can activate dopamine D_1_ receptor on dorsal motor nucleus of the vagus (DMV) neurons to regulate gastric motility in rats (Anselmi et al., 2017). However, the pathogenesis by which dopaminergic neuron degeneration in the SN results in constipation remains unclear.

Constipation may occur as a result of prolonged colonic transit and/or anorectal dysfunction in PD patients (Stocchi and Torti, 2017). Lower GI tract motility is controlled by the sacral parasympathetic nucleus (SPN) and ENS. The alterations associated with constipation and defecatory dysfunctions have been investigated in the ENS and SPN of PD patients and animal models with PD. Several studies have revealed the existence of neuronal degeneration with alpha-synuclein deposition in the SPN and ENS of patients with PD (Bloch et al., 2006; Gold et al., 2013). Moreover, imaging studies have revealed decreased expression of intestinal acetylcholinesterase in patients with early stages of PD (Gjerloff et al., 2015; Fedorova et al., 2017). But, some studies have reported that the choline acetyltransferase (ChAT) expression in the proximal colon of rats with nigrostriatal denervation by 6-OHDA did not change (Colucci et al., 2012). Of note, the pathology of Lewy bodies has been observed in colonic and rectal cholinergic neurons, suggesting that cholinergic pathways are involved in colorectal dysmotility in PD (Sharrad et al., 2013). Our previous studies have demonstrated that reduced cholinergic neurons, decreased D_1_ and increased D_2_ receptor expression in the DMV, and decreased gastric ACh levels are involved in delayed gastric emptying in 6-OHDA rats (Zheng et al., 2011, 2014; Cai et al., 2013; Wang et al., 2016). However, the effects of SN destruction on colorectal motility and cholinergic neurons in the SPN have not been determined.

The aim of the present study was to examine the effect of central dopaminergic denervation by injection 6-OHDA into the bilateral SN, on colorectal cholinergic contractive activity in rats. *In vivo* whole GI and colorectal transit was evaluated by barium meal assay and bead expulsion test. Electrical field stimulation (EFS) induced and bethanechol chloride induced cholinergic contractions were recorded *in vitro* from longitudinal muscle of colorectal preparations. The ACh level, expression of ChAT protein, and muscarinic receptor (MR) of the colorectum were assessed by ultra- performance liquid chromatography-tandem mass spectrometry (UPLC-MS/MS), immunohistochemistry and western blot, respectively. Moreover, the vasoactive intestinal peptide (VIP) content and expression of neural nitric oxide synthase (nNOS) in the colorectum was also detected by radioimmunoassay and western blot, respectively. This study will reveal the pathogenesis of constipation and provide evidences regarding the potential drug target for constipation in PD.

## Materials and Methods

### Animals

Male Sprague-Dawley rats (200–230 g) were purchased from the Laboratory Animal Services Center of Capital Medical University, Beijing, China. All animals were housed under a 12-h/12-h light-dark cycle at 22 ± 1°C with free access to food and water. All experiments were performed in accordance with the guidelines established by the National Institutes of Health and were approved by the Animal Care and Use Committee of Capital Medical University, Beijing, China.

### 6-Hydroxydopamine Rats

The procedures for preparing 6-OHDA rats have been described previously (Tian et al., 2008; Zheng et al., 2014; Zhang et al., 2015). Rats were anesthetized with a mixture of xylazine and ketamine (13 and 87 mg/kg body weight, respectively; intraperitoneal) and placed on a Kopf stereotaxic instrument. A 6-OHDA solution (4 μg in 2 μL of 0.9% saline containing 0.05% ascorbic acid) was bilaterally injected into the SN at the following coordinates (mm): anteroposterior,–5.6; mediolateral, ± 2.0; and dorsoventral, –7.5. Control rats underwent sham stereotaxic surgery and were injected with 0.2% ascorbic acid/saline. Subsequent experiments were performed at 6 weeks after 6-OHDA administration.

### Whole Gastrointestinal Transit Time and Colorectal Transit Time

Barium meal (1.5 mL, barium sulfate) or phenol red (1.5 mL, 5% w/v suspended in 0.5% methylcellulose) was administered to each rat after an 18-h fast by gastric lavage. The whole-GI transit time was calculated as the interval between gavage and the time of the first observance of white feces (barium meal) or red feces (phenol red).

Colorectal transit time was measured using a bead expulsion test according to our previous study (Liu et al., 2018). Rats were anesthetized with isoflurane, and then a 6-mm glass bead was inserted into the colorectum (4?cm proximal to the anus) using a plastic pasteur pipette that was lightly lubricated with lubricating jelly. The time until bead expulsion was measured. The colorectum in rats is similar to the rectosigmoid in humans and is located between the distal colon and the rectum. The colorectum (approximately 1–2 cm in length) is adjacent to the lymph node, which is typically situated 1–2 cm from the anus (Zhang et al., 2008, 2010).

### Fecal Water Content

The method was described in our previous study (Zhang et al., 2015). The fecal water content was calculated according to the equation: Fecal water content = 100% × (wet weight – dry weight)/wet weight.

### Motility Recording

#### *In vivo* Recording

The method used in this study has been described previously (Zheng et al., 2014; Liu et al., 2018). Rats were anesthetized with a mixture of xylazine and ketamine (13 and 87 mg/kg body weight, respectively; intraperitoneal). Body temperature was monitored with a rectal probe and maintained at 37 ± 1°C using a homeothermic pad placed under the animal. A midline incision was made in the skin and muscle layers of lower abdomen to expose the colon. Next, strain gauge transducers (WS100, Xin Hang Xing Ye Tech. Co., Beijing, China) were implanted on the serosal side of colorectum to record the longitudinal and circular muscle contractions, respectively. Then, a preamplifier and Lab Chart software (PowerLab, ADInstruments, Sydney, Australia) were used to record the colonic movement. The area under the curve of 8 min was taken to calculate to tension of contraction (mg^∗^s).

#### *In vitro* Recording

According to the methods in our previous study (Zhang et al., 2012, 2015; Liu et al., 2018), longitudinal muscle strips from the colorectum were immersed in an organ bath with 5 mL of Krebs-Henseleit solution (K-HS) and then mounted vertically under an initial tension of 1 g. The K-HS (composition in mM: NaCl, 117; KCl, 4.7; NaHCO_3_, 24.8; KH_2_PO_4_, 1.2; MgCl_2_ ⋅ 6H_2_O, 1.2; CaCl_2_ ⋅ 2H_2_O, 2.5; and glucose, 11.1; pH, 7.4) was maintained at 37°C and oxygenated with 95% O_2_ and 5% CO_2_. The strips were equilibrated for at least 1 h. The contraction reached a stable level and maintained a stable plateau for 30 min before the application of drugs or EFS stimulation. EFS (2 ms, 50 V, 5 Hz) was applied from an electrical stimulator (BL-420F, Chengdu Techman Software Co., Chengdu, China) (Liu et al., 2018). The tension was recorded with an isometric force transducer (MLT0201/RAD, ADInstruments, Spain) and digitized by a bridge amplifier (ML228; ADInstruments, Sydney, Australia). The area under the curve of 8 min was taken to calculate to tension of contraction (mg^∗^s).

### Tissue Preparation

The colorectum were opened along the mesenteric border and pinned flat in a SYLGARD-lined Petri dish containing ice-cold oxygenated K-HS solution. The muscular layer of the colorectum was carefully microdissected with fine forceps under a dissection microscope. The tissue was immediately snap-frozen in liquid nitrogen for protein extraction and quantification.

### Acetylcholine and Vasoactive Intestinal Peptide Measurements

The methods of ACh have been partially described in our previous studies (Zheng et al., 2014). ACh levels were measured by ultra- performance liquid chromatography-tandem mass spectrometry (UPLC-MS/MS). Each sample (30 mg) was homogenized in 300 μL of 12% aqueous formic acid. The homogenates were ultrasonically dissociated with 1.5 mL of reconstitution solvent (acetonitrile: methanol: formic acid, 750:250:2) for 2 min and centrifuged at 12,000 rpm for 15 min at 4°C. The supernatant was evaporated to dryness, redissolved with 300 μL of reconstitution solvent, and centrifuged at 3,000 rpm for 5 min at 4°C. The supernatant was used immediately for UPLC-MS/MS (Key Laboratory of Radiopharmaceuticals, Ministry of Education, College of Chemistry, Beijing Normal University).

VIP content was measured with commercial radioimmunoassay kits (KIPL 0300, HY-091; Beijing Sino-UK Institute of Biological Technology, Beijing, China).

### Western Blot Analysis

Frozen muscle tissue or spinal cord (L6-S1) tissue was homogenized in ice-cold lysis buffer (Applygen Technologies Inc., Beijing, China) with protease inhibitor cocktail (Roche, Switzerland). Equal amounts (50 μg) of extract were separated by 10% SDS-PAGE and transferred to a polyvinylidene fluoride membrane (Millipore, United States) at 4°C. Non-specific binding sites were blocked with 10% non-fat milk in TBS (20 mM Tris-Cl, pH 7.5, containing 0.15 M NaCl and 2.7 mM KCl) for 1 h at room temperature. The membrane was incubated with the primary antibodies listed in Table 1 overnight at 4°C. After washes with TBST, the membrane was incubated with the appropriate secondary antibodies goat anti-rabbit IgG (1:10,000, 611-132-122, Rockland, United States) or sheep anti-mouse IgG (1:10,000, 610-632-002, Rockland, United States) for 2 h at room temperature. The membrane was washed in TBST and visualized with an Odyssey Infrared Imager (LI-COR, NE, United States). The integrated intensity of the bands was analyzed with Odyssey software (version 1.2).

**TABLE 1:** Primary antibodies.

Antigen	Host species	Dilution		Source/Catalog no.
		Immunofluorescence	Western blot	
TH	Mouse	1:5,000	N/A	Sigma/T1299
ChAT	Goat	1:200	1:1,000	Millipore/AB144P
ChAT	Rabbit	N/A	1:1,000	Proteintech/20747-1-AP
nNOS	Rabbit	N/A	1:1,000	Cell signaling/4236
M1	Rabbit	1:200	1:200	Alomone/AMR-001
M2	Mouse	1:200	1:1,000	ThermoFisher/MA3-044
M3	Rabbit	1:200	1:500	Abcam/87199
M3	Rabbit	1:200	1:200	Alomone/AMR-006
GAPDH	Rabbit	N/A	1:10,000	Sigma/G9545

*TH, tyrosine hydroxylase; ChAT, choline acetyltransferase; nNOS, neuronal nitric oxide synthase; M, muscarinic receptor; GAPDH, glyceraldehyde-3-phosphate dehydrogenase; N/A, not applicable.*

### Immunohistochemistry

For immunohistochemistry, rats were anesthetized with 10% chloral hydrate (4 mL/kg, intraperitoneal), and were perfused through the left ventricle with 200 mL saline, and then with 200 mL 4% paraformaldehyde solution in 0.01 M phosphate-buffered saline (PBS; pH 7.4). The brains and spinal cords were immediately removed, and immersed in the 4% paraformaldehyde for 24 h at 4°C. After dehydration with 15 and 30% sucrose in 0.01 M PBS, serial coronal frozen sections of the SN and spinal cord were cut at a 20 μm thickness with a cryostat microtome (Leica CM1850, St. Gallen, Switzerland). Tissue sections were air dried overnight at room temperature. As described in our previous study (Zheng et al., 2011), brain sections and spinal sections were immersed in citrate buffer (0.01 M, pH 6.0) for antigen retrieval and were then treated with 3% H_2_O_2_ for 10 min. After an overnight incubation at 4°C with primary antibodies (Table 1), the sections were incubated with a mouse biotinylated anti-goat IgG antibody or a rabbit biotinylated anti-goat IgG antibody (ZSGB-BIO, Beijing, China) for 10 min. Next, the reaction products were developed using diaminobenzidine chromogen (ZSGB-BIO, Beijing, China). Following the reaction, the sections were dehydrated in alcohol, cleared in xylene and overlaid with a coverslip.

L6-S1 spinal segments were collected for ChAT immunohistochemistry in the SPN region (Vizzard et al., 2000; Naitou et al., 2016). The immunolabeled cells in bright-field microscopic images were counted manually by two independent investigators. One of every 3 coded sections was collected for ChAT immunohistochemistry. All cell counts were location-matched between control and 6-OHDA rats (five animals per group). The counts of ChAT-immunoreactive (IR) cells are presented as the average number of cells per section (mean ± SEM).

### Immunofluorescence

For immunofluorescence staining, protocols were the same as previously described (Zhang et al., 2015; Liu et al., 2018). Colons were rinsed with PBS, frozen, and embedded in optimum cutting temperature medium (McCormick, St. Louis, Missouri). Tissues sections at 7 μM were fixed in ice-cold acetone for 15 min. After washing with PBS 3 times for 5 min, the sections were incubated with 5% donkey serum for 30 min. Then, the sections were incubated with primary antibodies (listed in Table 1) overnight at 4°C, followed by 2 h of incubation with the secondary antibodies donkey anti-mouse IgG (1:1,000, A11055, Invitrogen, United States) at room temperature. The negative control was performed by omitting the primary antibody.

### Statistical Analysis

The results are presented as the means ± SEM, with “n” referring to the number of different animals from which tissues were collected. All statistical analyses were performed using one-way ANOVA followed by the Newman-Keuls test or Student’s paired or unpaired *t*-tests (GraphPad Prism 6.0, San Diego, CA, United States). *P*-values less than 0.05 were considered to be significant.

## Results

### Outlet Obstruction Constipation in 6-Hydroxydopamine Rats

Similar to our previous study, the DA content in the SN and striatum was substantially reduced in 6-OHDA rats (Supplementary Figures 1A–C). There was no significant difference in daily food or water intake between the two groups (Supplementary Figures 1D,E). However, decreased daily fecal pellet elimination and fecal water content were observed in 6-OHDA rats (Supplementary Figures 1F,G).

**FIGURE 1:**
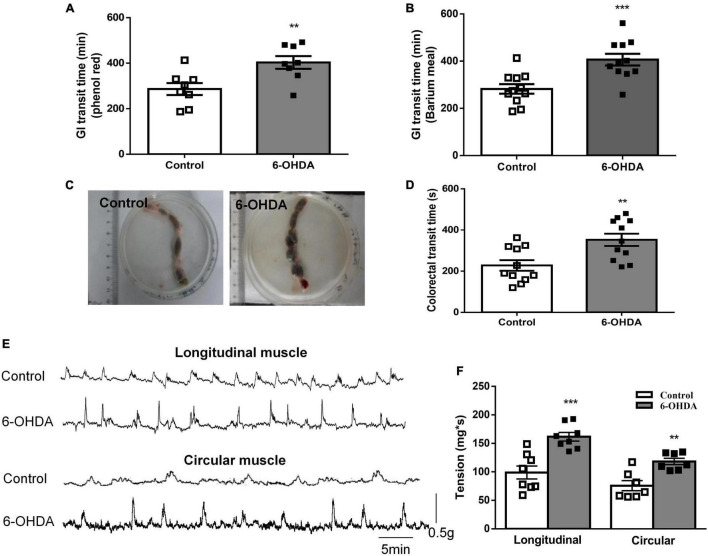
Outlet obstruction constipation in 6-OHDA rats. **(A)** Whole GI transit time of phenol red (*n* = 8). **(B)** Whole GI transit time of barium meal (*n* = 11). **(C)** Fecal retention in the colorectum of 6-OHDA rats. **(D)** Colorectal transit time using a bead expulsion test (*n* = 11). **(E)** Representative tracings of the colorectal spontaneous contraction of control and 6-OHDA rats. **(F)** Bar charts show the contractive tension of colorectum (*n* = 8). **P < 0.01, ***P < 0.001.

The whole GI transit time of phenol red and barium meal was increased by 49.6% (422 ± 24 vs. 282 ± 20 min, Figure 1A, *n* = 8, *P* < 0.01) and 40.9% (403 ± 28 vs. 286 ± 26 min, Figure 1B, *n* = 11, *P* < 0.001) in 6-OHDA rats compared to control rats, respectively. Fecal retention in the colorectal was observed in 6-OHDA rats (Figure 1C). Moreover, the colorectal transit time was significantly prolonged in 6-OHDA rats (control, 352 ± 29 s; 6-OHDA, 228 ± 26 s, Figure 1D, *n* = 11, *P* < 0.01). *In vivo* motility recording showed that the contractive tension of both longitudinal muscle and circular muscle was obviously enhanced in the colorectum of 6-OHDA rats (Figures 1E,F, *n* = 8, *P* < 0.001). These results suggested that 6-OHDA rats exhibited outlet obstruction constipation.

### Effect of Electrical Field Stimulation on Colorectal Motility in 6-Hydroxydopamine Rats

**FIGURE 2:**
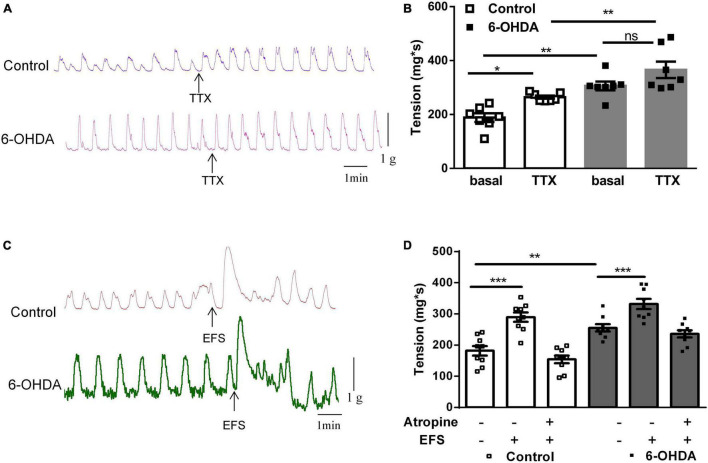
Effect of tetrodotoxin (TTX) and electrical field stimulation (EFS) on the contraction of colorectum. **(A)** Representative tracings of *in vitro* colorectal contraction when pretreatment with TTX (1 μM). **(B)** Bar charts show the contraction of colorectal (*n* = 7). **(C)** Representative tracings of EFS-invoked colorectal contraction. **(D)** Bar charts show the contraction of the colorectum in response to EFS alone or in combination with the muscarinic receptor antagonist atropine (1 μM) (*n* = 9). *P < 0.05, **P < 0.01, ***P < 0.001.

To explore the mechanism of enhanced colorectal contraction in 6-OHDA rats, the longitudinal muscle strips were isolated for recording spontaneous contractile activity *in vitro*. Isolated colorectal strips tracings showed that the contraction of was remarkably increased in 6-OHDA rats (Figures 2A,B, *n* = 7, *P* < 0.01). To discriminate between neuronal and muscular mechanisms, the muscle strips were pretreated with tetrodotoxin (TTX; 1 μM), a blocker of neuronal sodium channels, to prevent neuronal regulation of muscular activity. Pretreatment with TTX markedly increased the contraction of colorectum in control rats (40%, from 188.5 ± 16.24 mg^∗^s to 264.4 ± 5.87 mg^∗^s, *P* < 0.05). However, TTX-induced contraction was only increased by 20% in 6-OHDA rats (from 306.1 ± 16.2 mg^∗^s to 365.7 ± 30.29 mg^∗^s, Figures 2A,B, *n* = 7). This result indicated that enteric neurons were involved in the colorectal dysmotility in 6-OHDA rats. The EFS was then used to further explore the enteric neuronal mechanism underlying colorectal dysmotility in 6-OHDA rats. EFS induced-contraction in the colorectum was increased by 59% in control (from 181.8 ± 15.23 to 289.3 ± 16.87 mg^∗^s, *P* < 0.005) and 30% in 6-OHDA rats (from 255.2 ± 11.57 to 332.2 ± 16.26 mg^∗^s, *P* < 0.005) (Figures 2C,D, *n* = 9). The increasing level of EFS- evoked contraction was less in 6-OHDA rats than that in controls. And, the effect of EFS was blocked by atropine, a MR antagonist (Figure 2D). Thus, cholinergic transmission was involved in EFS-induced colorectal contraction, which was diminished in 6-OHDA rats.

### Effect of Bethanechol Chloride on Colorectal Motility in 6-Hydroxydopamine Rats

**FIGURE 3:**
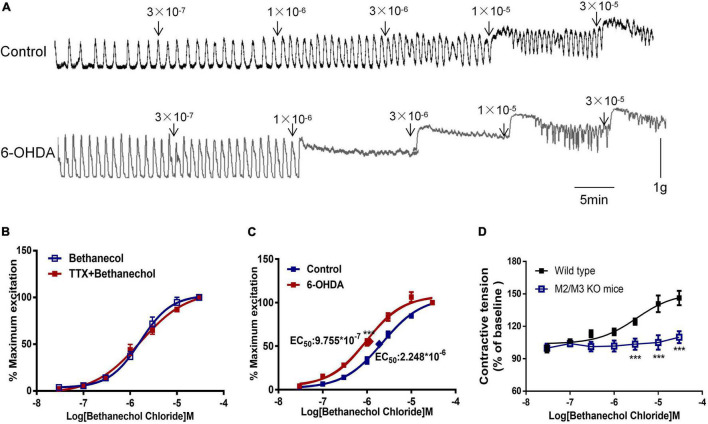
Effect of bethanechol chloride on colorectal contraction. **(A)** Representative recordings show the effect of bethanechol chloride on colorectal muscle strips from control and 6-OHDA rats. **(B)** Concentration response curve of bethanechol chloride alone and in the presence of TTX (1 μM) in control rats (*n* = 6). **(C)** Dose-response curve of bethanechol chloride in the colorectum of control and 6-OHDA rats (*n* = 6). **(D)** Concentration-response curve of bethanechol chloride in M_2_R and M_3_R knockout (KO) mice (*n* = 5). **(B–D)** Two strips per rat. ***P < 0.001.

Bethanechol chloride, a selective MR agonist, dose-dependently increased colorectal contractions (Figure 3A). Pretreatment with TTX did not affect the concentration-response curve of bethanechol chloride, indicating that enteric neurons were not involved in the bethanechol chloride-induced excitatory effect (Figure 3B). The concentration-response curve of bethanechol chloride was shifted leftward in 6-OHDA rats. The EC_50_ was 8.859 × 10^–7^mol/L in 6-OHDA rats compared with 2.379 × 10^–6^ mol/L in controls (Figure 3C, *n* = 6, *P* < 0.005), reflecting enhanced MR reactivity in the colorectum of 6-OHDA rats. Bethanechol chloride increased colorectal contractions in wild-type mice to a maximum of 140% of the baseline value in a concentration-dependent manner, but failed to increase colorectal contractions in M_2_R and M_3_R knockout (KO) mice (Figure 3D, *n* = 5, *P* < 0.005), suggesting that M_2_R and M_3_R predominantly mediated the effect of bethanechol chloride.

### Acetylcholine and Vasoactive Intestinal Peptide Content, Protein Expression of Choline Acetyltransferase, Neural Nitric Oxide Synthase, and Muscarinic Receptor in Colorectum

**FIGURE 4:**
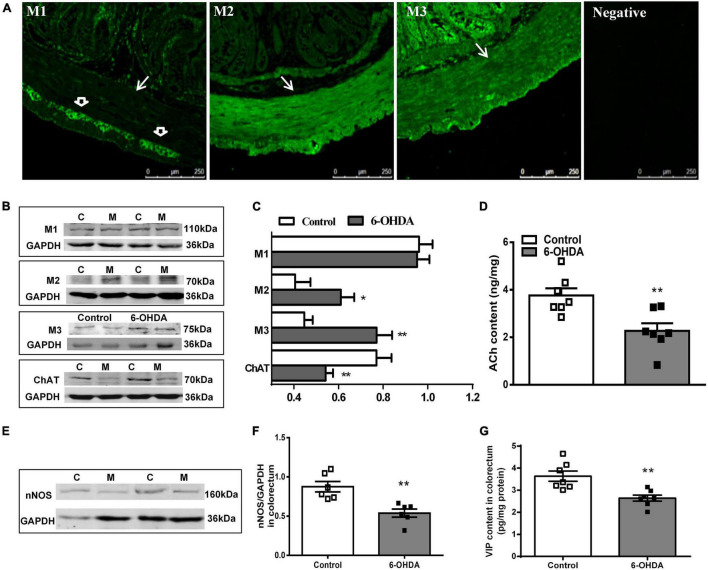
**(A)** Distribution of M_1_R, M_2_R, M_3_R in the colorectum of control rat. The arrow indicates the muscular layer; the open arrowhead indicates the myenteric plexus. Negative control without the primary antibodies. **(B,C)** Representative western blot and bar charts show levels of the M_1_R, M_2_R, M_3_R, and ChAT protein in the colorectum of control (C) and 6-OHDA rats (M) (*n* = 7). Glyceraldehyde-3-phosphate dehydrogenase (GAPDH) was used as an internal control. **(D)** ACh content in the colorectal muscularis externa (*n* = 7). **(E,F)** Representative western blot and histogram show levels of the nNOS protein in the colorectum (*n* = 6). **(G)** VIP contents in the distal colonic and colorectal muscularis externa (*n* = 7). *P < 0.05, **P < 0.01.

The distribution of M_1_R, M_2_R, and M_3_R in the colorectum of rats was measured by immunofluorescence. As shown in Figure 4A, M_2_-IR and M_3_-IR were abundantly expressed in the muscular layer, but M_1_-IR was predominant in the myenteric plexus, not the muscular layer. No immunofluorescence was detected in the negative control. Furthermore, an obvious increase in the M_2_R and M_3_R protein expression was detected in the colorectal muscularis externa in 6-OHDA rats, whereas M_1_R did not change (Figures 4B,C, *n* = 7).

The protein level of ChAT (decreased by 32%, *P* < 0.01) and nNOS (decreased by 40%, *P* < 0.01) was markedly reduced in the colorectal muscularis externa of 6-OHDA rats (Figures 4B,C,E,F). Similarly, ACh and VIP content were significantly decreased (Figures 4D,G, *P* < 0.01).

### Expression of Choline Acetyltransferase in the Sacral Parasympathetic Nucleus

**FIGURE 5:**
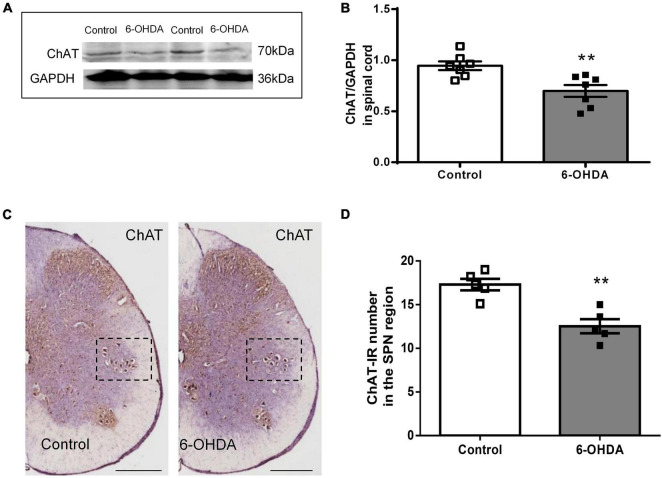
Expression of choline acetyltransferase (ChAT) in the sacral parasympathetic nuclei (SPN). **(A,B)** Representative Western blot and bar charts show the levels of the ChAT protein in the L6-S1 spinal segment tissue (*n* = 7). GAPDH was used as an internal control. **(C)** The expression of ChAT in the SPN region (black frame). Scale bar, 500 μm. **(D)** The ChAT-IR number in the SPN region (*n* = 5). **P < 0.01.

As shown in Figure 5, western blot showed that ChAT protein level in L6-S1 spinal segments were reduced from to 0.95 ± 0.04 to 0.70 ± 0.06 in 6-OHDA rats (Figures 5A,B, *n* = 7, *P* < 0.05). Furthermore, ChAT-IR neurons were detected in the SPN. The number of ChAT-IR cells was decreased approximately 18.5% in the SPN region in 6-OHDA rats compared to control rats (Figures 5C,D, *n* = 5, *P* < 0.05).

## Discussion

**FIGURE 6:**
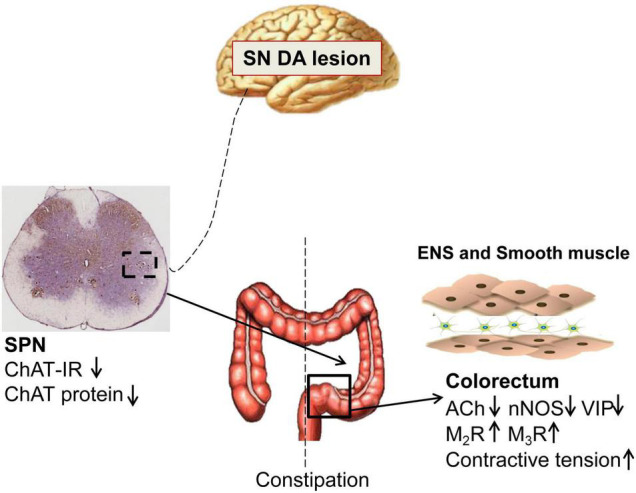
Schematic model of the pathogenesis of constipation in 6-OHDA rats. The number of cholinergic neurons and expression of choline acetyltransferase (ChAT) protein in the sacral parasympathetic nuclei (SPN) was decreased in rats with central nigrostriatal dopaminergic denervation. Decreased expression of nNOS and VIP in the colorectum might be primarily responsible for the increased colorectal contractile tension and outlet obstruction constipation. Meanwhile, the impaired cholinergic transmission and upregulation of M2/M3 might also partially involve in the colorectal dysmotility.

PD patients exhibit significantly slow transit in the rectosigmoid segment and whole colon, and anorectal dysfunction (Sakakibara et al., 2003; De Pablo-Fernandez et al., 2019; Tan et al., 2021). Both slow-transit and outlet obstruction constipation, in isolation or in combination, have been observed in PD patients (Sakakibara et al., 2003; Borghammer et al., 2016). However, the underlying mechanisms are unclear. Our data indicated that 6-OHDA rats exhibited outlet obstruction constipation characterized by increased colorectal transit time and enhanced colorectal contractive tension, which are partly similar to the phenomena in clinical studies. The present study demonstrated that central nigrostriatal dopaminergic denervation (one of the main pathologic hallmarks of PD), was associated with decreased cholinergic neurons in the SPN, decreased ACh, VIP and nNOS, but upregulated M_2_R and M_3_R in the colorectum, resulting in colorectal dysmotility, which contributed to outlet obstruction constipation (Figure 6).

Pellet propulsion in the colorectum is a coordinated neuromuscular process regulated by neurotransmitters in the ENS. This process involves oral contraction mediated by excitatory transmitters such as ACh and substance P (SP), and caudad relaxation mediated by inhibitory neurotransmitters such as vasoactive intestinal peptide (VIP), pituitary adenylate cyclase-associated peptide (PACAP), and nitric oxide (Grider et al., 1998). In the present study, the sustained and enhanced contraction in the colorectum of 6-OHDA rats may lead to difficulty in fecal propulsion. Blocking enteric neuronal activity with TTX increased the colorectal motility, suggesting that the inhibitory effect of ENS on colorectal contraction occurs in physiological condition. Similar results have also been observed in the cat intestine, mouse intestine and rat distal colon (Wood, 1980; Li et al., 2006; Zhang et al., 2012). However, the effect of ENS was reduced in the colorectum of 6-OHDA rats. To verify whether the enhanced *in vivo* colorectal contraction might depend on underlying alterations of enteric neurotransmission, we focused on the colorectal neurotransmitters. Our results showed that EFS-induced cholinergic contraction was diminished in the colorectum of 6-OHDA rats. When possible alterations of intestinal cholinergic pathways are considered in PD, the available evidence is not consistent. Systemic administration of the selective dopaminergic neurotoxin MPTP induces loss of dopaminergic neurons in both the SN and colon in mice and non-human primates (Tian et al., 2008). The MPTP animals showed no differences in cholinergic or VIP in ENS (Anderson et al., 2007; Chaumette et al., 2009). Functionally, MPTP-treated mice show a transient increase in colon motility, but no obvious changes in stool frequency (Anderson et al., 2007). Another PD animal model with 6-OHDA-induced right medial forebrain bundle (MFB) lesions has been found to exhibit significantly increased VIP and reduced nNOS expression without altered ChAT expression in the distal ileum and proximal colon (Colucci et al., 2012), whereas EFS evoked-ACh release are significantly reduced in colon (Fornai et al., 2016). Of note, PD patients display a significant loss of acetylcholinesterase signal in the small intestine (Gjerloff et al., 2015; Fedorova et al., 2017) and reduced VIP-IR neurons in the colonic submucosal plexus (Giancola et al., 2017). Consistent with these findings, our previous study have shown a drastic decrease ChAT protein expression and ACh content in the gastric corpus and duodenal mucosa of 6-OHDA rats (Zheng et al., 2014; Yan et al., 2021). We suppose that the impairment of cholinergic neurotransmission may lead to decrease in the role of EFS in the colorectum of 6-OHDA rats. In addition, we also found that the protein expression of nNOS and VIP content was decreased in the colorectum. Combined with the weakened of TTX-increased contraction in colorectum of 6-OHDA rats, which demonstrated that diminished inhibition (decreased nNOS and VIP), might be primarily responsible for the increased colorectal contractile tension.

In addition, we also observed an enhancement of spontaneous contractive tension and bethanechol chloride-induced myogenic response in the colorectum of 6-OHDA rats. In the present study, TTX did not affect the dose-response curve of bethanechol chloride, indicating that MR in the muscular layer was directed activated by bethanechol chloride. Three subtypes of MR are distributed throughout the GI tract (Harrington et al., 2010). M_2_R and M_3_R are the main subtypes mediating cholinergic contractions (Eglen, 2001; Kondo et al., 2011). Similarly, our result showed that bethanechol chloride was not able to promote colonic contraction in the M_2_R and M_3_R KO mice. Therefore, we examined the expression of M_2_R and M_3_R in the colorectal muscular layer. Both of M_2_R and M_3_R was indeed upregulated in the colorectum of 6-OHDA rats, occurring as a compensatory response to the impaired cholinergic neurotransmission, which may partially involve in colorectal dysmotility. Fornai et al. (2016) has reported the upregulated M_2_R and M_3_R in colon of the unilaterally MFB lesion model. But, they did not investigate the spontaneous contraction. In the present study, enhanced, but not weakened, colorectal contractive tenison resulted in the difficult of fecal propulsion. Overall, it is conceivable that outlet obstruction constipation in 6-OHDA rats depends, at least in part, on the impaired cholinergic neurotransmission in colorectum.

The colorectum is innervated by parasympathetic pelvic nerves (Naitou et al., 2016), which arise from the SPN (Vizzard et al., 2000). Moreover, the ENS is also regulated by the preganglionic nerve terminals of the SPN. SPN pathology has also been reported in PD patients (Bloch et al., 2006). Activation of cholinergic neurons in the SPN promotes colorectal propulsive contractions in rats (Naitou et al., 2016). Therefore, decreased cholinergic neurons and reduced expression of ChAT protein in the SPN may reduce the release of neurotransmitters in ENS and slow down fecal propulsion in 6-OHDA rats. Our previous study has revealed the presence of dopaminergic nigrostriatal projections to the hypothalamic paraventricular nucleus (PVN) and locus coeruleus (LC) (Wang et al., 2014). As reported, the PVN participates in the network through projections to pontine nuclei, such as the Barrington nuclei, which have monosynaptic connections with the sacral parasympathetic centers (Nuding and Nadelhaft, 1998; Valentino et al., 2000; Vizzard et al., 2000). Therefore, neuronal degeneration in the SN may indirectly affect the cholinergic neurons in the SPN. However, the regulatory mechanism needs to be further elucidated.

Overall, enhanced, but not weakened, colorectal contractive tenison has been exhibited in 6-OHDA rats. In conclusion, central nigrostriatal dopaminergic denervation is associated with decreased cholinergic neurons in SPN, decreased expression of nNOS and VIP in the colorectum, which might be primarily responsible for the increased colorectal contractile tension and outlet obstruction constipation. Meanwhile, the impaired cholinergic transmission and upregulation of M2/M3 might also partially involve in the colorectal dysmotility. The present study provides more clues for the pathogenesis and evidences regarding the potential drug target for constipation in PD.

## Data Availability Statement

The original contributions presented in the study are included in the article/Supplementary Material, further inquiries can be directed to the corresponding author/s.

## Ethics Statement

The animal study was reviewed and approved by Animal Care and Use Committee of Capital Medical University, Beijing, China.

## Author Contributions

J-XZ designed the research project. X-LZ and X-HZ performed the research and analyzed the data. XY, L-FZ, X-YF, C-ZL, Z-SQ, and YZ provided technical support. X-LZ and J-XZ wrote the manuscript. All authors critically reviewed and revised the manuscript.

## Conflict of Interest

The authors declare that the research was conducted in the absence of any commercial or financial relationships that could be construed as a potential conflict of interest.

## Publisher’s Note

All claims expressed in this article are solely those of the authors and do not necessarily represent those of their affiliated organizations, or those of the publisher, the editors and the reviewers. Any product that may be evaluated in this article, or claim that may be made by its manufacturer, is not guaranteed or endorsed by the publisher.

## Supplementary Material

Supplementary Figure 1 Characterization of the 6-OHDA rats. **(A)** TH-immunoreactive (IR) neurons in the substantia nigra (SN). (a,c) Are low-magnification views of the SN. (b,d) Are higher-magnification views of the SN within the frames of (a,c). Scale bar, 100 μm. **(B,C)** Dopamine (DA) content in the SN (B, *n* = 7) and striatum (C, *n* = 9). **(C,D)** Food and water consumption of control and 6-OHDA rats (*n* = 9). **(E,F)** Daily fecal production and fecal water content between control and 6-OHDA rats (*n* = 11). ^∗∗^*P* < 0.01, ^∗∗∗^*P* < 0.001.

Supplementary Data Sheet 1 The raw data supporting the conclusion of the article.

## Abbreviations

6-OHDA: 6-hydroxydopamine
TH: Tyrosine hydroxylase
SN: Substania nigra
PD: Parkinson’s disease
ChAT: choline acetyltransferase
ACh: acetylcholine
DA: dopamine
NE: Norepinephrine
MR: muscarinic receptor
nNOS: Neural nitric oxide synthase
VIP: Vasoactive intestinal peptide
SPN: sacral parasympathetic nuclei
ENS: enteric nervous system.
