# Supplementary figures and images for: Enhanced Contractive Tension and Upregulated Muscarinic Receptor 2/3 in Colorectum Contribute to Constipation in 6-Hydroxydopamine-Induced Parkinson’s Disease Rats
Source: Front Aging Neurosci. 2021 Dec 23;13:770841. doi: 10.3389/fnagi.2021.770841 (PMC8733788; doi:10.3389/fnagi.2021.770841)

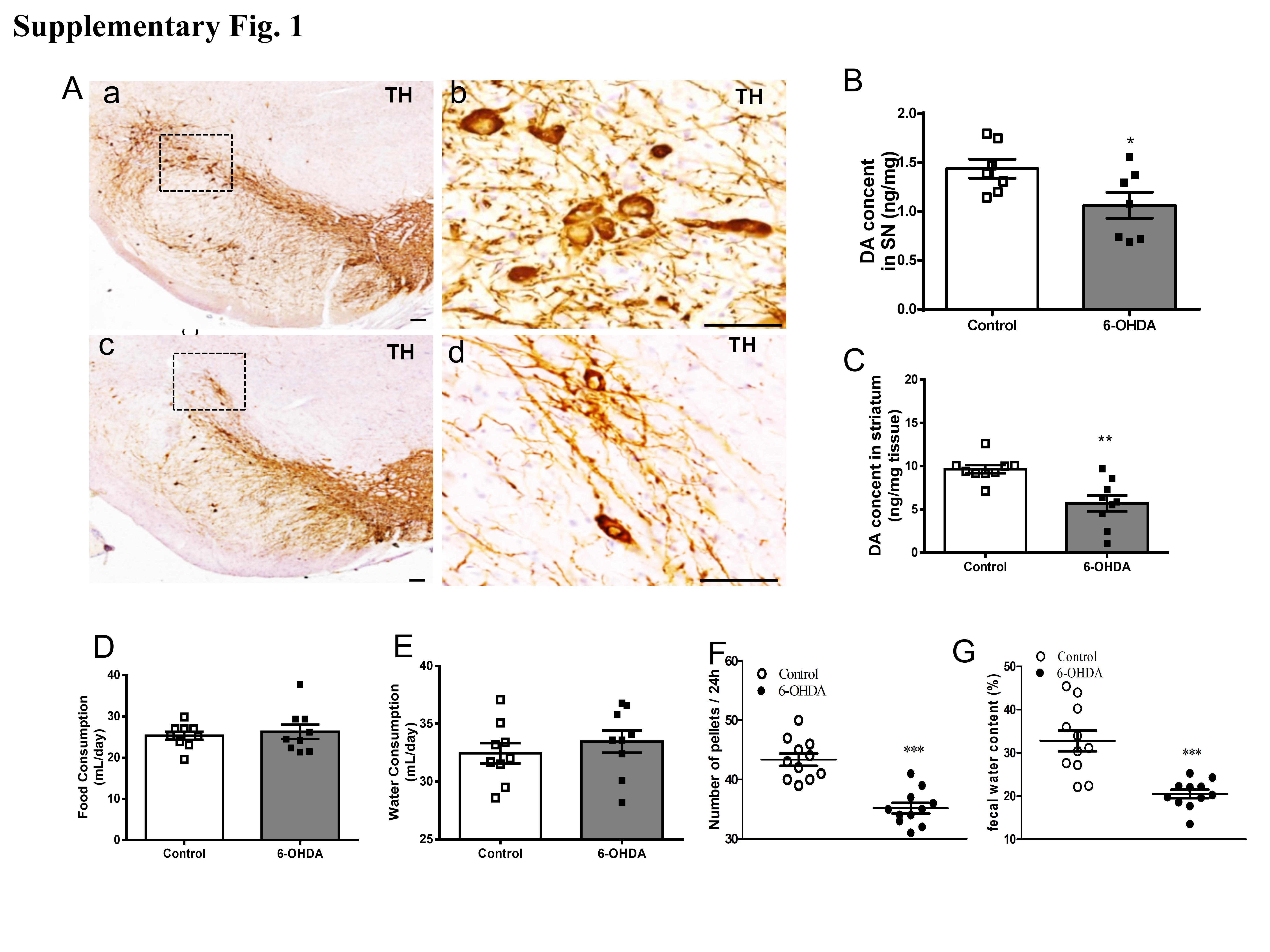

Supplement: Supplementary Figure 1 — Characterization of the 6-OHDA rats. (A) TH-immunoreactive (IR) neurons in the substantia nigra (SN). (a,c) Are low-magnification views of the SN. (b,d) Are higher-magnification views of the SN within the frames of (a,c). Scale bar, 100 μm. (B,C) Dopamine (DA) content in the SN (B, n = 7) and striatum (C, n = 9). (C,D) Food and water consumption of control and 6-OHDA rats (n = 9). (E,F) Daily fecal production and fecal water content between control and 6-OHDA rats (n = 11). ∗∗P < 0.01, ∗∗∗P < 0.001. [file Image_1.JPEG]
